# BARIMEP: A TOOL FOR TRAINING BARIATRIC SURGERY PATIENTS

**DOI:** 10.1590/0102-672020230002e1720

**Published:** 2023-03-31

**Authors:** Carolina Mocellin GHIZONI, Ligia de OLIVEIRA-CARLOS, Giorgio Alfredo Pedroso BARETTA, Arieli Luz Rodrigues BARETTA, Maria Paula Carlin CAMBI, Antonio Carlos Ligocki CAMPOS

**Affiliations:** 1 Universidade Federal do Paraná, Postgraduate Course in Clinical Surgery – Curitiba (PR), Brazil; 2 Clínica Dr. Giorgio Baretta – Curitiba (PR), Brazil

**Keywords:** Mindful Eating, Feeding Behavior, Bariatric Surgery, Comer com Atenção Plena, Comportamento Alimentar, Cirurgia Bariátrica

## Abstract

**BACKGROUND:**

Bariatric surgery patients have symptoms such as “plugging.” Therefore, a possible good way to avoid these eating discomforts, typical of the early period after bariatric surgery, is to educate the patient. The Mindful Eating (ME) consists of paying attention to physical signs of hunger and satiety and developing awareness of emotional triggers related to food. In addition, conscious food choices reflect positively on the speed of chewing at mealtime.

**AIMS:**

Due to the difficulties that patients reported during consultations to controlling their bad eating habits and the lack of tools to help the bariatric patient change eating habits, we elaborated “BariMEP: A Mindful Eating Placemat for bariatric surgery patients.”

**METHODS:**

The BariMEP was written by the multidisciplinary bariatric team based on a study by Russell et al. and ME principles in order to help bariatric patients pay attention to what and how they eat at each meal.

**RESULTS:**

The BariMEP has some instructions based on Mindful Eating principles: get your seat at the table; do not distract yourself; before starting to eat, try breathing sometimes; recognize the internal hunger and satiety cues; let the fork rest at each bite and chew a lot; pay attention to the smell and taste; and be as present as possible at this time with nonjudgment.

**CONCLUSIONS:**

For the first time, a tool has been developed with the aim of preparing the patient for bariatric surgery. Since the BariMEP is easy to teach and cheap, we suggest that the BariMEP be included in the bariatric surgery protocol.

## RESUMO

**RACIONAL:**

Pacientes operados de cirurgia bariátrica apresentam sintomas como “entalo”. Portanto, uma possível maneira de evitar esses desconfortos alimentares, típicos do período inicial após a cirurgia bariátrica, é educar o paciente. O Mindful Eating (ME) consiste em avaliar aos sinais físicos de fome e saciedade e desenvolver a consciência dos gatilhos emocionais relacionados à alimentação. Além disso, as escolhas alimentares conscientes refletem positivamente na velocidade da mastigação durante as refeições.

**OBJETIVOS:**

Devido às dificuldades, que os pacientes relataram durante as consultas, para controlar os maus hábitos alimentares e a falta de ferramentas para ajudar o paciente bariátrico na mudança, elaboramos o “BariMEP: um jogo americano de comer com atenção plena para pacientes de cirurgia bariátrica”.

**MÉTODOS:**

O BariMEP foi escrito pela equipe multidisciplinar bariátrica com base no estudo de Russell et al. e nos princípios do Mindful Eating, para ajudar os pacientes submetidos a cirurgia bariátrica a prestar atenção ao que e como comem em cada refeição.

**RESULTADOS:**

O BariMEP possui algumas instruções baseadas nos princípios do Mindful Eating: Sente-se à mesa; não se distraia; antes de começar a comer, respire algumas vezes; reconheça os sinais de fome e saciedade; deixe o garfo descansar a cada mordida e mastigue bastante; preste atenção ao aroma e sabor; esteja o mais presente possível neste momento e sem julgamentos.

**CONCLUSÃO:**

Pela primeira vez, foi desenvolvida uma ferramenta com o objetivo de preparar o paciente para a cirurgia bariátrica. Como o BariMEP é fácil de ensinar e barato, sugerimos que o BariMEP seja incluído no protocolo de cirurgia bariátrica.

## INTRODUCTION

Obesity is considered a pandemic disease, and its prevalence is increasing. According to the World Health Organization, the prevalence of obesity has been increased almost three times since 1975 and it affects about 600 million people worldwide. This condition significantly increases the risk of heart disease, stroke, type 2 diabetes, and cancer, among others^20^. It is the result of a complex junction of genetic, behavioral, psychological, and environmental factors. The optimal treatment for obesity should include diet, physical activity, and changing habits. However, it is difficult to induce and maintain weight loss in individuals with obesity^4^.

Since conventional treatments do not have lasting results, bariatric surgery is usually recommended. Candidates for bariatric surgery are patients with a body mass index (BMI) greater than 40 kg/m^2^ or a BMI greater than 35 kg/m^2^ associated with comorbidities (e.g., arterial hypertension, dyslipidemia, type 2 diabetes, and sleep apnea)^4^.

Bariatric surgery is considered the most effective treatment for severe obesity because it is associated with permanent weight loss, resolution of comorbidities, and improved life expectancy^2^. However, maladaptive eating habits after surgery typically represent a continuation or recurrence of preoperative eating patterns^21^. The masticatory profile of the obese person is significantly altered in relation to people without obesity, and this is one of the greatest challenges of post-bariatric adaptation. These patients have symptoms such as “plugging,” defined as food getting stuck in the small opening of the pouch with epigastric discomfort^21^.

In addition, to prevent nausea and vomiting, it is important to advise patients to respect the diet evolution in relation to the consistency and volume of food, and again to eat slowly, to chew food to a pureed consistency, and to stop eating as soon as they feel full^12^.

Therefore, a possible good way to avoid these eating discomforts, typical of the early period after bariatric surgery, is to educate the patient before weight loss surgery. International guidelines suggest that in the preoperative period, patients should take control of their food behavior: eating less quantity of food, decreasing the size of the bites and sips, and chewing food slowly and extensively, with the aim of decreasing caloric intake, optimizing the digestive process, ensuring adequate surgery recovery, and adapting a new post-bariatric lifestyle^21^.

In the preoperative period of bariatric surgery, the psychologist must investigate several aspects of the patient’s life, not only to determine their readiness for the operation but also to educate them regarding the changes implied through it^4^.

The difficulty for humans to modify behavior, especially eating behavior, has been studied^3^. Problematic eating behaviors, such as binge and emotional eating, have been characterized in obesity^5^. The performance of routine activities on “autopilot” can cause the patient to eat on impulse and without control^8^.

According to Chacko et al., a significant reduction in emotional eating was observed with a mindfulness-based intervention in bariatric patients after surgery. The authors suggested further exploration in longer term studies using mindfulness-based interventions for weight control after bariatric surgery^2^.

The Mindful Eating (ME) approach consists of paying attention to physical signs of hunger and satiety and developing awareness to physical, cognitive, socioenvironmental, and emotional triggers related to food. In addition, conscious food choices reflect positively on the speed of chewing at mealtime^3^. There is also a positive effect of ME on emotional eating and the reduction of the eating reflex in response to external stimuli^3^.

Schnepper et al. evaluated the effects of a short weight-loss intervention that combines mindfulness techniques with prolonged chewing^18^, and their study suggested that these two techniques might help with eating behavior at mealtime.

No tool has been identified which uses ME techniques to help bariatric patients change eating behaviors and maybe avoid plugging after bariatric surgery.

Habit is a type of automatism, and the behavior can occur without the person being aware of this occurrence^8^. Due to the lack of tools to help the bariatric patient change eating habits, a multidisciplinary team in a private bariatric clinic elaborated a “BariMEP: A Mindful Eating Placemat for bariatric surgery patients” (BariMEP) based on difficulties that patients reported during consultations to control their bad eating habits. The BariMEP was based on a study by Russell et al. which developed a placemat to preparation for inpatient colonoscopies. The authors concluded that the educational placemat was easy to introduce and was helpful in empowering patients with easy-to-follow instructions. Furthermore, the financial cost of implementing this project was low^17^.

In Russel et al.’s^17^ study, the placemat was printed on A3 paper to be used during the meal, thus reminding the patient to practice ME and thus encouraging them for new habits and not repeating the old automatic pattern^10^.

The aim of this study was to share a new tool that could be helpful to better prepare the patient for surgery and prevent the patient from some adverse effects after bariatric surgery.

## METHODS

The BariMEP was developed based on the study entitled “Patient-centered approaches to targeting incomplete bowel preparations for inpatient colonoscopies” by Russell et al.^17^. The BariMEP was written by a multidisciplinary bariatric team and printed on A3 paper with the ME principles that could help the patients undergoing surgery. The study was approved by the Ethical Committee of the Institution (nº 12476019.3.0000.0020).

## RESULTS

**Mindful Eating Tool: BariMEP, a Mindful Eating Placemat**

**Figure 1.**
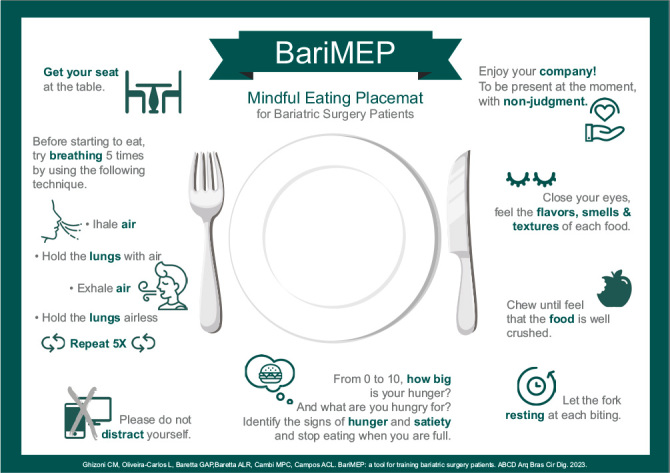
BariMEP: A Mindful Eating Placemat for training bariatric surgery patients.

The BariMEP contains the most important points of ME principles^10,13,16^ which are described in a simple and clear graphical presentation (Figure 1). This tool can help the multidisciplinary team explain ME to bariatric patients before the operation and support them during meals.

## DISCUSSION

During the preoperative period, patients should be educated about the importance of the ME approach and encouraged to pay attention to what and how they eat at each meal. The BariMEP has some instructions based on ME principles^9–11,13,16^:

1. **“Get your seat at the table”:** It is important to sit with attention at the table during the meal and to be present at this moment. Usually, when people do not sit and pay attention at this moment, they probably eat more than needed. Mindfulness is characterized by continually attending to one’s moment-by-moment experiences, thoughts, and emotions with an open, nonjudgmental approach^1^.
2. **“Do not distract yourself”:** If the patient is distracted by a television or smartphone, he or she will not pay attention at mealtime. Patients tend to respond to diverse stimuli and lack of awareness in eating actions, repeating inappropriate behavior patterns^12^. People should make sure that their focus is only on what they are eating and how they are doing it.
3. **“Before starting to eat, try breathing sometimes by using the following technique. Inhale air by nose, hold the lungs with air, exhale air by mouth, hold the lungs airless”**: Breathe deeply to relax and to be able to eat slowly and mindfully at this moment^8^. A study by Hendrickson and Rasmussen^7^ demonstrated that a brief training in ME was associated with a reduction in impulsive patterns in the choice of foods. Overeating may suggest that environmental or external cues prevail, and the person acts on autopilot to eat^8^. ME can be useful to interrupt these automatic, nonconscious external influences and can help change eating behaviors^7^ being effective in reducing disordered eating^15^.
4. **“Think about how hungry you are”:** After sitting down at the table, people should think about their level of hunger and what they feel like eating. The plate must be set with portions consistent with their hunger. This command is for people to turn their attention to their feelings at that moment, recognizing internal hunger and satiety cues^14^.
5. **“Let the fork rest at each bite”:** They should eat appreciating to slow the eating. The performance of routine activities on “autopilot” can cause the patient to eat on impulse and without control. For this reason, some authors have investigated tools that could help the patient practice new behaviors in order to make them acquire new eating habits^12^.
6. **“Chew a lot before swallowing”:** The purpose of asking the person to chew until the food is ready to be swallowed is to stimulate them to be aware of this moment, as well as to notice the difference in some foods that have a different texture. Moreover, this strategy is extremely useful for patients who are in the postoperative period of bariatric surgery, who need to chew their food well to avoid vomiting and plugging^2,18^. With more awareness at meals, it is possible to eat more slowly and improve chewing, avoiding the plugging episodes that are common after bariatric surgery^2,18^. These plugging episodes are very common postoperative bariatric surgery and can bring a lot of discomfort to patients^3^.
7. **“Pay attention to the smell and taste. Notice how you are feeling about them”:** People must enjoy the food. To feel all that food can bring, explore how enjoyable and the pleasure of each taste is a huge experience that unfortunately we are losing with the automatism of daily life. In addition, eating slowly, paying attention to how the body is responding, and identifying the emotional eating as a stress response are fundamental for the physical and mental well-being of patients^3,19^.
8. **“Enjoy your company and be as present as possible at this time”:** Mindfulness is a practice of being present at the moment, nonjudgment, identifying the signs of hunger and satiety, and stopping eating when you are full^4^.

## CONCLUSIONS

For the first time, a tool has been developed with the aim of preparing the patient for bariatric surgery by bringing focus to mealtime and emphasizing this moment, what food is being eaten, the amount of each portion, and the speed of chewing. It is known that changing habits takes time and needs to be practiced many times, so a BariMEP could be helpful to bariatric patients in improving their eating habits and making healthier choices.

Since the BariMEP is easy to teach and cheap, we suggest that clinical research using the BariMEP should be undertaken because we believe that it could be a useful tool to be included in the prebariatric surgery protocol.
